# Study of the role of leukocyte telomere length-related lncRNA NBR2 in Alzheimer’s disease

**DOI:** 10.18632/aging.206107

**Published:** 2024-09-16

**Authors:** Wenjie Li, Haoyan Chen, Xiaofan Yuan, Qi Yao, Mingjiong Zhang

**Affiliations:** 1 Department of Geriatrics, The First Affiliated Hospital of Ningbo University, Ningbo 315000, China; 2 Department of Geriatrics, Jiangsu Key Laboratory of Geriatrics, The First Affiliated Hospital of Nanjing Medical University, Nanjing 210000, China; 3 Department of Radiology of the Second Affiliated Hospital of Nanjing Medical University, Nanjing 210011, China

**Keywords:** Alzheimer’s syndrome, leukocyte telomere length, mendelian randomization analysis, lncRNA NBR2, GJA1

## Abstract

Alzheimer’s Syndrome (AD) is a neurodegenerative disease that is prevalent in middle-aged and elderly people. As the disease progresses, patients gradually lose the ability to take care of themselves, which brings a heavy burden to the family. There is a link between leukocyte telomere length (LTL) and cognitive ability. To search for possible pathogenic mechanisms and potential therapeutic agents, we demonstrated a causal link between LTL and AD using Mendelian randomization analysis (MR). The expression of the target gene NBR2 and the downstream mRNA GJA1 and GJA1-related genes, pathway enrichment, and association with immune cells were further explored. Using the gene cluster-drug target interaction network, we obtained potential therapeutic drugs. Our study provides evidence for a causal link between AD and LTL, suggesting medicines that may treat and alleviate AD symptoms.

## INTRODUCTION

Alzheimer’s syndrome (AD) is a slow-onset and progressive brain disease. Over time, the disease progresses to severe memory problems, eventually causing patients to lose the ability to perform daily tasks [1]. AD is also one of the most common age-related neurodegenerative diseases, affecting approximately 6.5 million people aged 65 and older in the U.S. [2]. AD is, therefore, a significant challenge for global healthcare. However, the mechanisms underlying the relationship between AD and aging are unclear.

Telomeres are known as the “mitotic clock” of cell life, and their length reflects the replication history and potential of cells [3]. As we age and the number of cell divisions increases, some of the genes that make up telomeres fail to replicate fully due to multiple cell divisions, and the cell terminates its function and no longer divides [4]. Thus, severely shortened telomeres indicate cellular aging [5]. Leukocyte telomere length (LTL) is a widely used biomarker, and in general, leukocyte telomere length reflects the state of senescence of the body’s immune cell-associated circulating cells [6]. It is unclear whether LTL is a risk factor for AD development.

Competitive endogenous RNA (ceRNA) networks, in which long non-coding RNAs (lncRNAs) function as endogenous ceRNAs to sequester microRNAs (miRNAs) and thereby enhance the expression of messenger RNAs (mRNAs), have been increasingly recognized for their significant contributions to the pathogenesis of Alzheimer’s disease (AD) and immune-inflammatory responses [7–9]. Among them, mir-19, a downstream miRNA of the LTL-associated lncRNA NBR2 [10], is mainly enriched in neural progenitor cells (NPCs) in hippocampal tissues, and its expression is down-regulated during neuronal development, particularly affecting neuronal cell migration [11]. mir-19-3p, the mature body of mir-19 [12], has been shown to alleviate amyloid β-induced nerve injury and thereby slow down the developmental process of AD [13].

Using two-sample Mendelian analyses, our study first explored the causal link between LTL and AD. We obtained the downstream miRNAs by intersecting the high lncRNA expression in various brain tissues and using TargetScan, followed by protein and gene level screening of NBR2-related genes to get GJA1. We further explored the function of GJA1 and its related genes. Finally, a novel drug-target interaction network framework was used to screen potential drugs to delay AD progression.

## MATERIALS AND METHODS

### Mendelian randomization analysis

LTL-related GWAS data were obtained from the UK Biobank, with 446,367 participants undergoing LTL measurements.

Data on genetic variants associated with AD were obtained from the GWAS database, ID number GCST90012877. p < 1e-8 was used as the genome-wide threshold. After screening with continuous instability and weak variable removal tools, 59 enetic instrumental variables (IVs) were finally obtained. We determined positive results and causal associations by the inverse variance weighted algorithm. The results of the MR-Egger algorithm were used as a test to assess heterogeneity. The MR egger_intercept algorithm was used to detect the data’s diversity and evaluate the result’s robustness. The “RCiros” package was used to visualize the chromosomal location of SNPs associated with IVs. The summary-data-based Mendelian randomization (SMR) was used to screen the eQTL corresponding to IVs and obtain the related genes [14] (https://cnsgenomics.com/software/smr/). The data on the expression of IVs-related genes in various brain tissues were obtained from the GTEx eQTL summarized data (https://www.gtexportal.org/home/eqtlDashboardPage).

### Application of interaction networks

The core gene NBR2 was obtained by taking the intersection of IVs-related genes from various brain tissues. lncRNA NBR2 expression in brain tissue was obtained from http://www.alzdata.org/. Downstream miRNAs and their maturation bodies were predicted using the LncRNA2Target V3.0 database (http://bio-computing.hrbmu.edu.cn/lncrna2target/). Corresponding mRNAs were expected from the TargetScan database (https://www.targetscan.org/vert_80/). The mRNAs with |Total context++ score|>0.6 were selected for subsequent analysis. Finally, NETWORK was used for the presentation.

### Functional and immunological enrichment of GJA1 and related genes

GSEA enrichment analysis of GJAI, KEGG pathway, and immune-related function was performed by “clusterProfiler.” Temporal Cortex data samples were divided into a high-expression group and a low-expression group according to the target gene GJA1. The CIBERSORT algorithm demonstrated the expression group and the ratio of immune genes between different groups. The correlation between lymphocyte subpopulations and GJA1 was compared between the differences between the two groups. The GJA1-related gene clusters were screened by Spearman analysis with -0.6 < cor < 0.8 and p < 0.001. Subsequently, we visualized the GO, KEGG, immune cell correlation, and chromosomal location distribution of GJA1-related genes.

### Screening of potential therapeutic drugs

Based on the Human Gene Interaction Network, we calculated the known drug’s relevant target of action, its own drug's appropriate target of action, and an AD-related gene called proximity, which was converted into a z-score. P-values corresponding to the significance of each drug were computed by randomly perturbing the global network 1000 times. Based on the topological nature of the network, we performed subnetwork module mining to cluster drug targets and disease genes in each module.

### Statistical analysis

We conducted a two-sample MR analysis using R software with the TwoSample MR and MR-PRESSO packages to explore the causal relationship between LTL and AD. The major method was random-effects inverse variance weighted (IVW), supplemented by MR Egger, weighted median, simple mode, and weighted mode. Heterogeneity was assessed using Cochran’s Q statistic (IVW) and Rucker’s Q statistic (MR Egger), with p > 0.05 indicating no heterogeneity. Horizontal pleiotropy was evaluated using the MR Egger intercept test and MR-PRESSO, with p > 0.05 indicating no pleiotropy. MR-PRESSO also identified outliers. A “leave-one-out” analysis examined the influence of individual SNPs on the causal relationship. The MR-PRESSO global test assessed horizontal pleiotropy (p > 0.05), and the distortion test identified outliers, which were excluded before reassessing causal estimates. The Cross-platform normalized expression level of different genes in Entorhinal Cortex, Hippocampus, Temporal Cortex, and Frontal Cortex was assessed statistically by Student’s T-test. Meanwhile, correlation between the target gene expressions was studied by using Pearson’s correlation. GO and KEGG analysis were performed to calculate enrichment p-values using hypergeometric distribution tests. P-values were set at 0.05 for statistically significant differences. Data analysis was done using R Foundation version 4.2.0.

## RESULTS

### Leukocyte telomere length and Alzheimer’s syndrome

**Figure 1:**
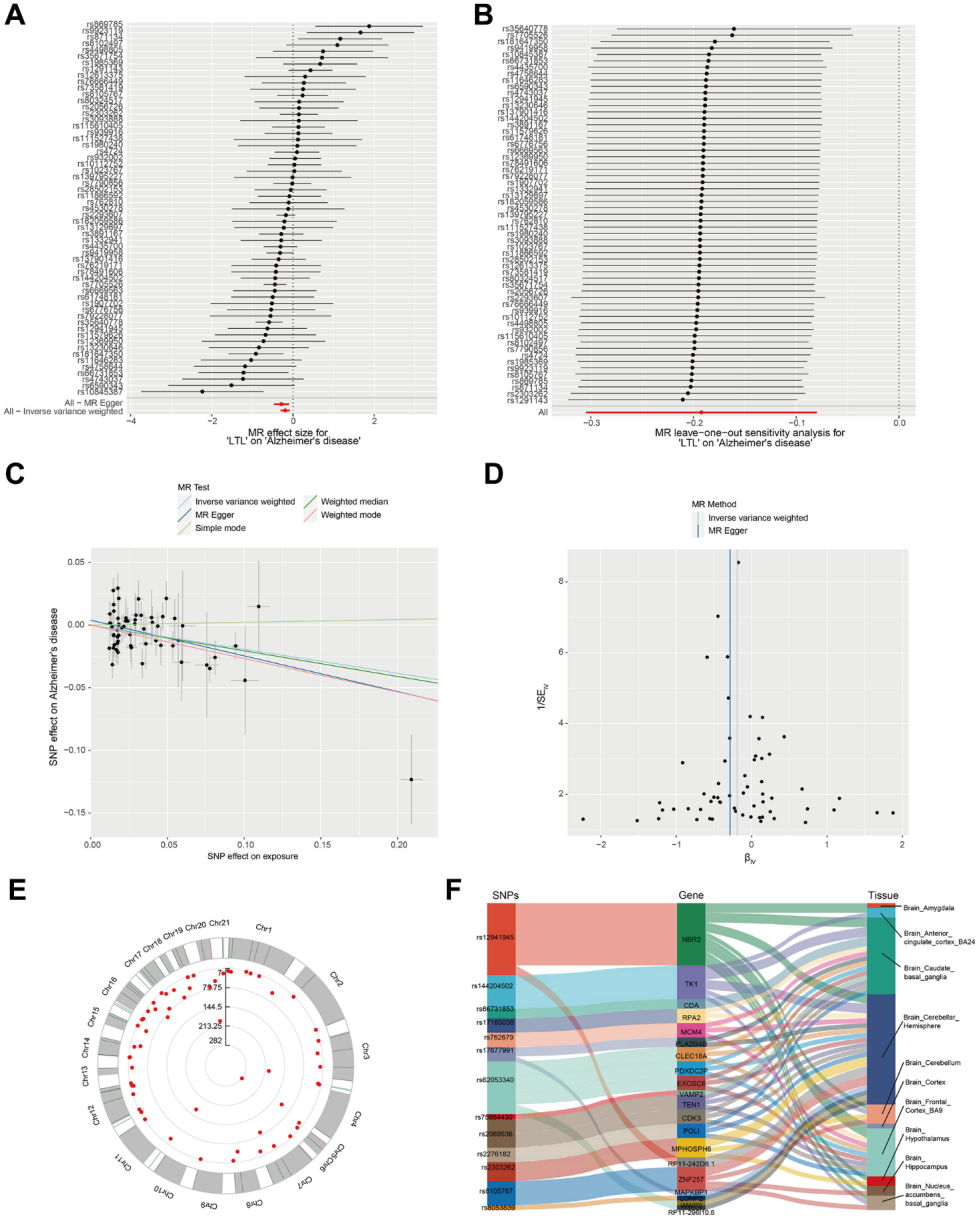
**Verification that leukocyte telomere length is correlated with Alzheimer's syndrome using Mendelian randomization.** (**A**) MR analysis for LTL on AD. (**B**) Sensitivity verification using leave-one-out analysis. (**C**) Scatterplot representing the causal link between LTL and AD. The horizontal axis reflects the genetic effect of each SNP on LTL. The vertical axis reflects the genetic impact of each SNP on the risk of developing AD. (**D**) Heterogeneity was assessed by IVW and MR-Egger tests. (**E**) Distribution of IVs associated with the location of SNPs on chromosomes. (**F**) Sankey diagram of SNP → gene ← tissue type.

GWAS data on leukocyte telomere length (LTL) associated with AD were obtained from open databases. The 59 SNPs strongly associated with AD after removing confounders and filtered by linkage disequilibrium and removing weak variables tools were treated as IVs (Figure 1A and Supplementary Table 1). Leave-one-out-analysis did not reveal abnormal SNP driving the association of IVs (Figure 1B). The scatter plots of the five MR analyses are shown in Figure 1C and Supplementary Table 2. The results of one of the IVW algorithms showed that LTL showed a negative correlation with AD. Heterogeneity was assessed by IVW and MR-Egger test, and p-value > 0.05 indicated no heterogeneity in the study (Figure 1D and Supplementary Table 3). Figure 1E demonstrates the location of IVs on chromosomes. As described in our previous analyses, we created a Sankey map of associated genes for IVs versus expression in various brain tissues to predict the expression of the genes related to IVs in brain tissues (Figure 1F and Supplementary Table 4).

### Analysis of genes corresponding to IVs

**Figure 2:**
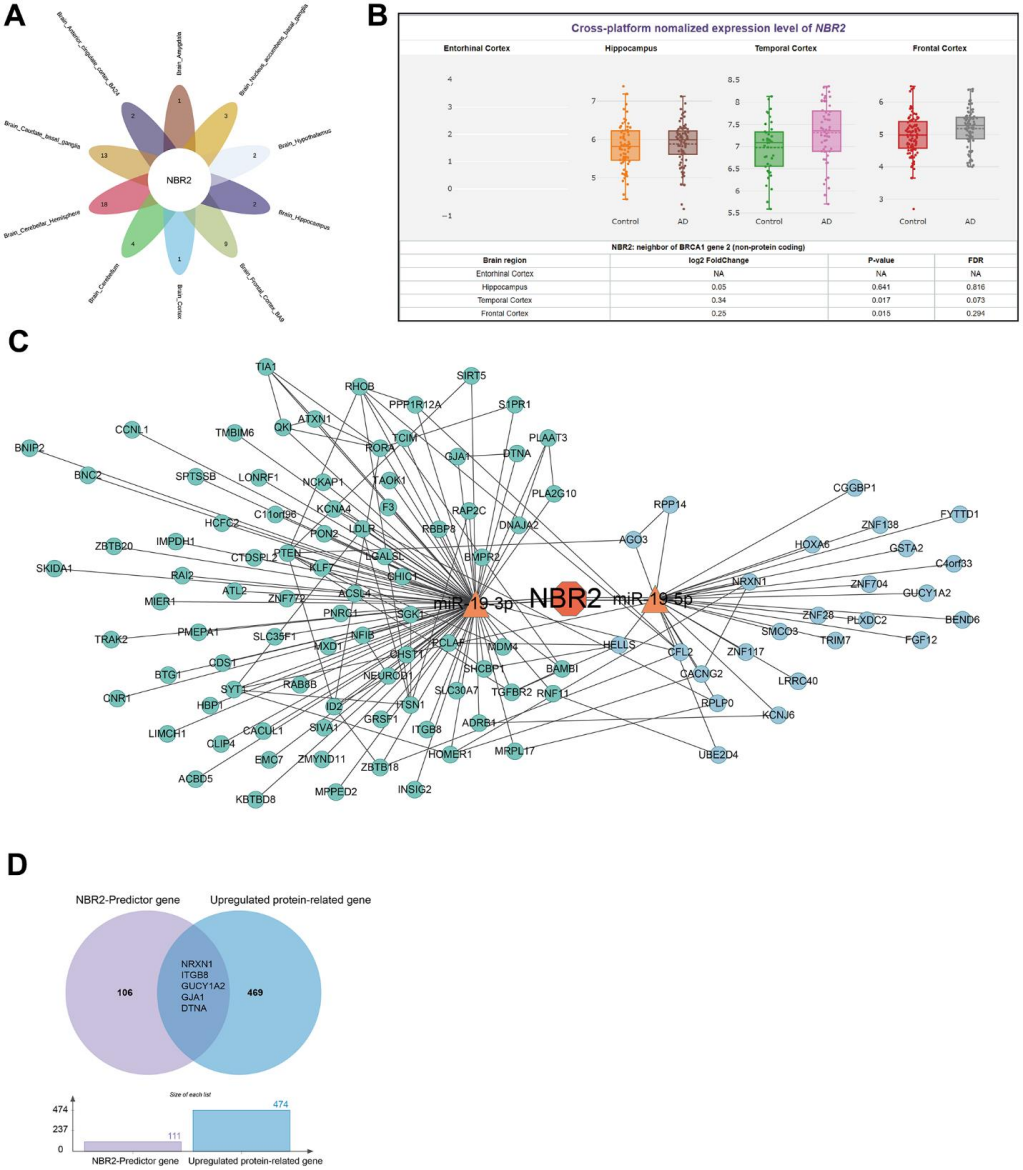
**Analysis of genes corresponding to IVs-associated SNPs.** (**A**) Venn diagram demonstrating that NBR2 is a core gene in various parts of the brain tissue. (**B**) Violin diagram demonstrating the expression of NBR2 in the significant components of the brain. (**C**) NETWORK diagram demonstrating miRNAs and mRNAs downstream of NBR2. (**D**) The Venn diagram demonstrates the high expression of 5 genes at both the protein and gene levels.

For brain amygdala, brain anterior cingulate cortex BA24, brain caudate basal ganglia, brain cerebellum, brain cortex, brain frontal cortex BA9, brain hypothalamus, brain hippocampus, and brain nucleus accumbens basal ganglia-related genes were taken to intersect, and the core gene NBR2 was finally obtained (Figure 2A). We used the information in the AIZ database to visualize the expression of NBR2 in primary brain tissues (Figure 2B). NBR2 was used as a lncRNA, and we used miRNA-lncRNA interactions analysis to obtain the possible downstream miRNAs, mir-19A. mir-19A has the maturation bodies of mir-19-3p and mir-19-5p. Subsequent analysis was carried out using miRNA target prediction (Supplementary Tables 5, 6). The miRNA target prediction software TargetScan was utilized to analyze the miRNA-mRNA network (Figure 2C). To ensure that the gene expression levels were consistent with the protein expression levels, we screened 469 genes related to highly expressed proteins based on the results of the data from Johnson ECB et al. (Supplementary Table 7). There were 106 NBR2-related gene sets (Supplementary Table 8). Taking the intersection of protein data with gene data yielded NRXN1, ITGB8, GUCY1A2, GJA1, and DTNA (Figure 2D).

### Differences in gene expression levels in primary brain tissues

**Figure 3:**
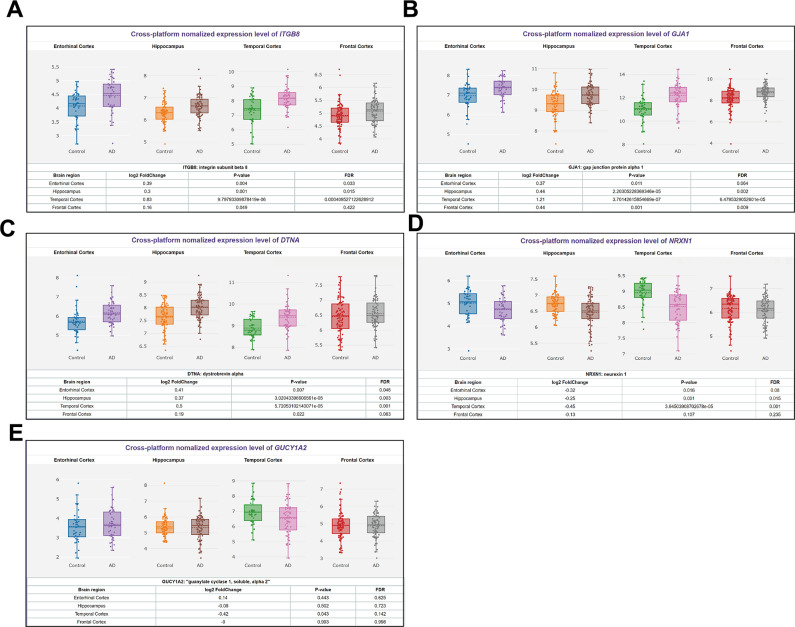
**Differences in expression levels of 5 predicted mRNAs in primary brain tissues of AD patients.** (**A**) ITGB8, (**B**) GJA1, (**C**) DTNA, (**D**) NRXN1, and (**E**) GUCY1A2 expression in Entorhinal Cortex, Hippocampus, Temporal Cortex, Frontal Cortex.

We used the AIZ database information to visualize the ITGB8 (Figure 3A), GJA1 (Figure 3B), DTNA (Figure 3C), NRXN1 (Figure 3D), and GUCY1A2 (Figure 3E) in Entorhinal Cortex, Hippocampus, Temporal Cortex, and Frontal Cortex Expression. Excluding NRXN1 and GUCY1A2, which had inconsistent gene and protein expression, we found that ITGB8, GJA1, and DTNA had the most significant expression differences in the Temporal Cortex. Among them, GJA1, with Log2FC = 1.21, was the most important and was selected as our subsequent target gene.

### Functional exploration of GJA1 at the single gene level

**Figure 4:**
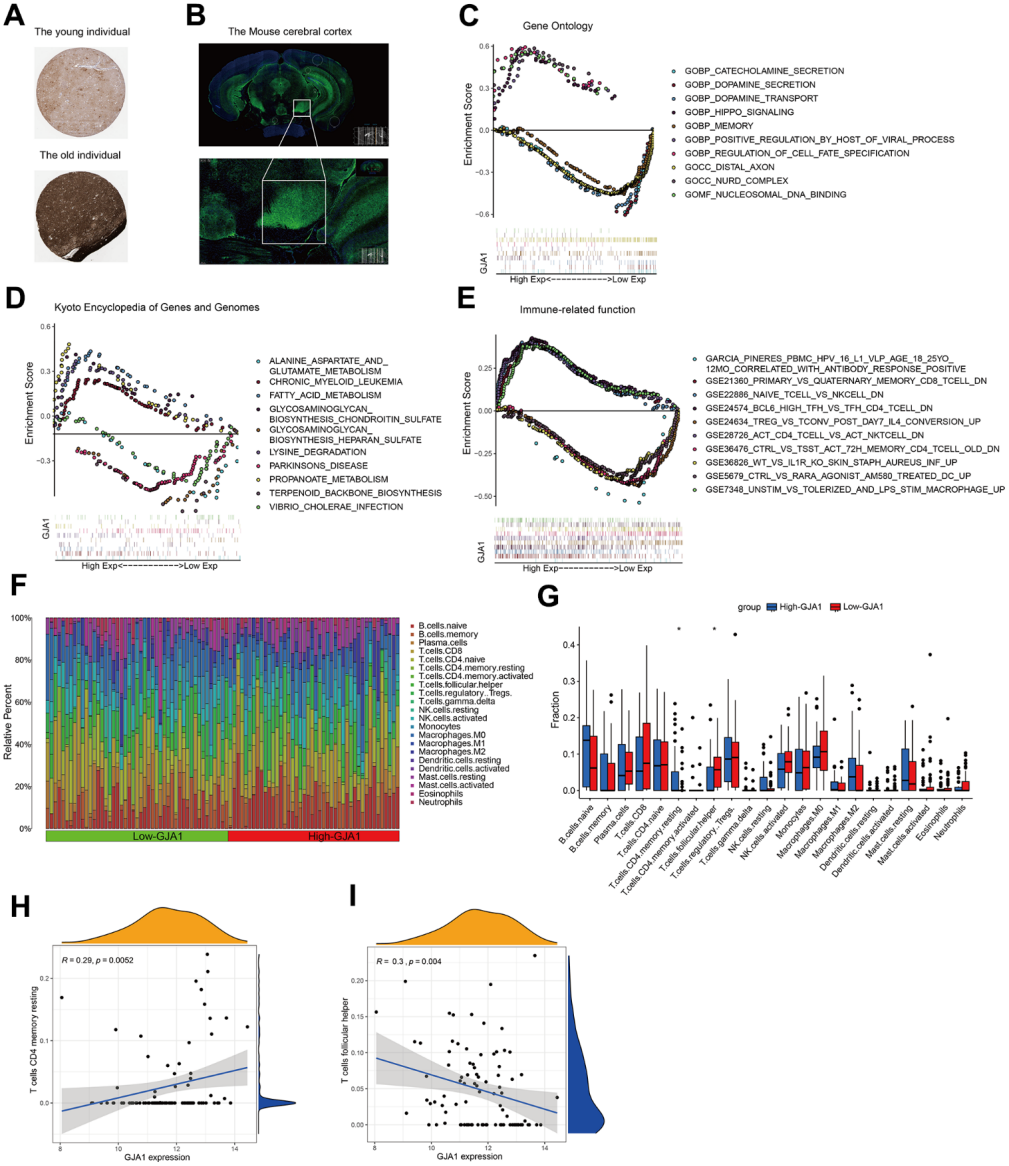
**Functional exploration of GJA1 at the single-gene level.** (**A**) Immunohistochemistry results demonstrating the expression of GJA1 in older populations. (**B**) Immunofluorescence demonstrating the enrichment of mouse brain tissue for GJA1 in the cortex. (**C**) GO analysis of GJA1. (**D**) KEGG analysis of GJA1. (**E**) Immune-related function analysis of GJA1. (**F**) Percentage of immune cells in the immune cell ratio between groups with high and low expression of GJA1 in Temporal Cortex. (**G**) Lymphocyte subpopulation occupancy in the two groups. (**H**, **I**) Correlation between lymphocyte subpopulation and GJA1 in the two groups.

To verify the expression of GJA1 in the brain tissues of young and old populations, we can get that GJA1 is more expressed in the brain tissues of geriatric populations using immunohistochemistry data from the HPA database (Figure 4A). Using the immunofluorescence results of mouse brain tissue, we can see that GJA1 is mainly enriched in the cortical part of the brain (Figure 4B). Single gene GO analysis (Figure 4C), KEGG analysis (Figure 4D), and Immune-related function analysis (Figure 4E) were performed for GJA1. The Temporal Cortex data samples were divided into high and low-expression groups according to the expression of the target gene GJA1, and the percentage of immune genes in the two groups was compared using the CIBERSORT algorithm (Figure 4F). Further comparing the two groups' lymphocyte subpopulations, we can get T cell CD4 memory resting accounted for more in the high-expression group, and T cell follicular helper accounted for more in the low-expression group (Figure 4G). Among them, T cell CD4 memory resting was positively correlated with the expression of GJA1 (R=0.29, p=0.0052), and T cell follicular helper was negatively correlated with the expression of GJA1 (R=0.3, p=0.004).

### Potential expanded functions of the GJA1

**Figure 5:**
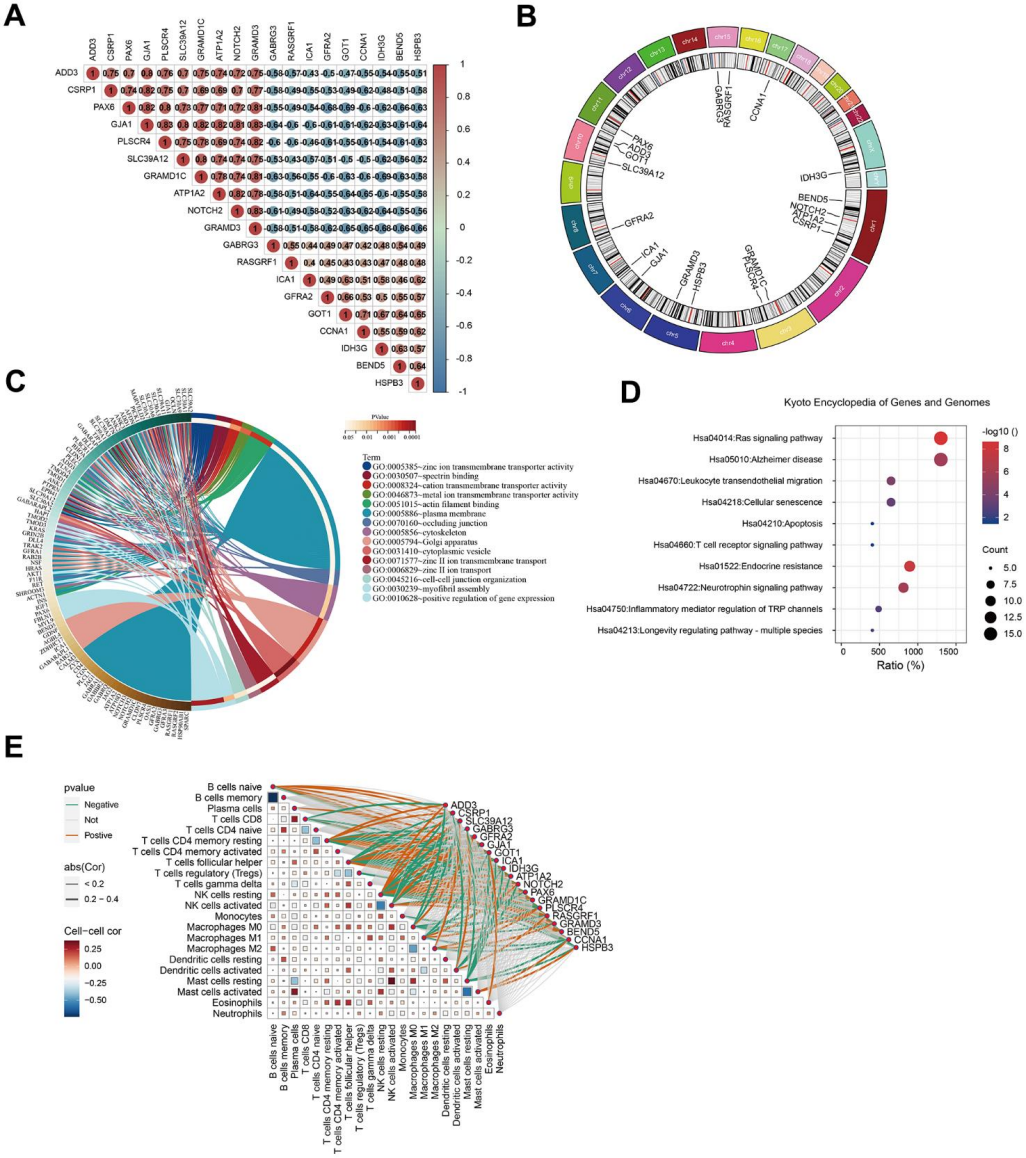
**Analysis of GJA1-related genes.** (**A**) Heatmap showing GJA1-related genes. (**B**) Chromosomal location distribution map of genes related to GJA1. (**C**) Circle map of GO analysis of the genes related to GJA1. (**D**) Circle map of KEGG analysis of the genes related to GJA1. (**E**) Correlation of the genes related to GJA1 with the immune cells.

We further explored the scalable functions of GJA1 in-depth and screened the genes closely related to GJA1 using Spearman analysis with p<0.001, -0.6<cor<0.8 (Figure 5A). Finally, 18 related genes were obtained, which were GABRG3, RASGRF1, IDH3G, BEND5, NOTCH2, ATP1A2, CSRP1, GRAMD1C, PLSCR4, GRAMD3, HSPB3, ICA1, GFRA2, SLC39A12, GOT1, ADD3, PAX6, CCNA1. Figure 5B shows the chromosomal location distribution of GJA1-related genes. GO analysis suggested that GJA1-related genes were mainly enriched on the plasma membrane (Figure 5C and Supplementary Table 9), and KEGG analysis suggested that they were mainly enriched on the Ras signaling pathway (Figure 5D and Supplementary Table 10). The linkage of GJA1-related genes with immune cells is shown in Figure 5E.

### GJA1-related gene set prediction of potentially targeted AD drugs

**Figure 6:**
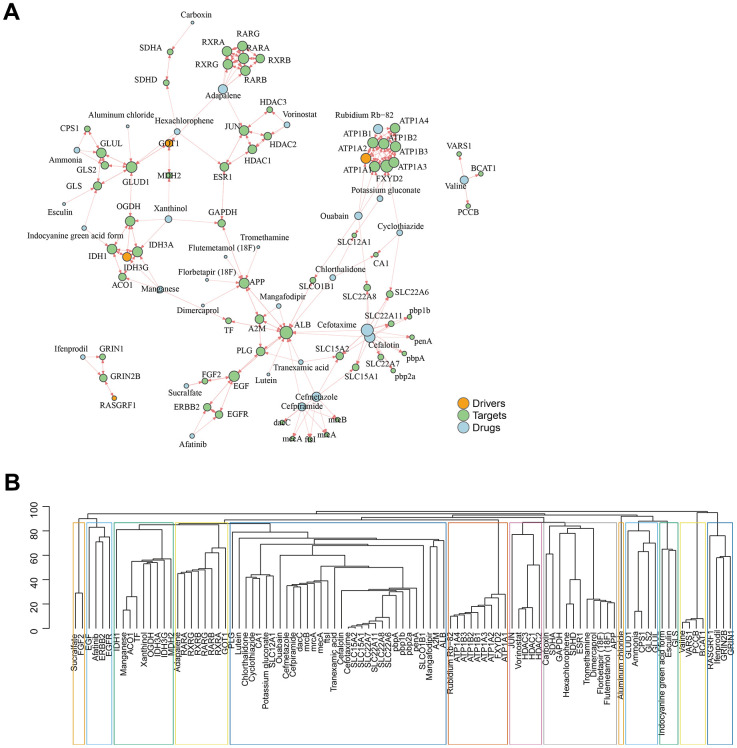
**GJA1-related gene set predicts subsequent AD-targeted drugs.** (**A**) Network diagram showing predicted drugs and targets. (**B**) Drug targets and disease genes are clustered in a module.

Based on the assumption that drugs are effective by targeting proteins within or near the corresponding disease module, we introduced an unsupervised and unbiased network framework to analyze the relationship between drugs and diseases. By linking the interactions network of drug targets with GJA1-related genes, we finally predicted that Adapalene, Rubidium Rb-82, Ammonia, Hexachlorophene, Vorinostat, Valine, Potassium gluconate, Ouabain Cyclothiazide, Chlorthalidone, Mangafodipir, Cefotaxime, Cefalotin, Tranexamic acid, Cefmetazole, and Cefpiramide are potential target drugs (Figure 6A). Based on the topological nature of the network, we then performed subnetwork module mining to cluster the drug targets and disease genes in each module (Figure 6B).

## DISCUSSION

LTL is commonly associated with aging, with data from a UK biobank of over 450,000 individuals suggesting a link between LTL and human health status [15]. Several studies have suggested a link between LTL and age-related cognitive decline in older adults. In a survey by Daniela et al., LTL shortening in AD patients appeared to arise from progressive telomere erosion, which may be associated with cognitive decline in the transition from aMCI to AD [16]. From another perspective, reduced LTL indicates active cell proliferation and may reflect the immune system's involvement in AD pathogenesis [17]. However, in some studies, this association was relatively minor or absent [18]. Based on this, we hypothesized that in peripheral leukocytes, telomere length is associated with an increased risk of age-related phenotypes. We then used Mendelian randomization analysis to explore the causal link between AD and LTL, filling in the gap at the SNP level.

80% of the human genome is non-coding RNAs involved in various biological functions [19]. There is evidence that miRNAs and lncRNAs are simultaneously engaged in AD ontogeny and development, including the formation and development of β-amyloid (Aβ) plaques, neuro progenitor fiber tangles, synapse loss, and neuronal death [20]. And they have specific temporal and spatial expression patterns. This suggests that LTL, our “aging clock,” is associated with lncRNAs, so we used the bioinformatics data from the AIZ database to select NBR2, a lncRNA with high expression of IVs-related genes in brain tissues, as the main entry point for our study. It interacts with AMPK and enhances AMPK activation under energy stress, thereby mediating cellular energy metabolism [21]. Currently, it is mainly used as a biomarker in hepatocellular carcinoma, colorectal cancer, thyroid cancer, and non-small cell lung cancer. Following this, downstream mature bodies mir-19-3p and mir-19-5p were obtained by miRNA-lncRNA interaction analysis. Downstream mir-19-3p can functionally inhibit protein translation of CCNA2 in the human body, thus affecting learning ability and memory in the brain [13].

NBR2, as a non-coding RNA, is not involved in protein translation, so we intersected its related gene data with data on highly expressed proteins in brain tissues and finally selected GJA1 as a subsequent target gene. GJA1, also known as connexin 43 (Cx43), functions as a connexin hemichannel and is involved in paracrine processes [22]. GJAI is predominantly expressed in mature astrocytes, and astrocyte gap junctions are critical for neuronal function. GJA1 was demonstrated to affect AD development by altering astrocyte function in a study by Yuji Kajiwara et al. This is consistent with our findings.

Disease development is often not limited to single-gene defects, and our study explores the role of related gene clusters in AD development centered on target genes and more comprehensively demonstrates the function of NBR2 in this context. Based on the coordinated interactions of the gene clusters, we used a drug-disease proximity measure to establish a gene-drug interaction network for predicting potential therapeutic drugs [23]. This method mainly uses the distance between the target gene corresponding to the drug and the target gene corresponding to the disease to indicate medicinal potential of medicines. The “proximal drugs” intervene in the endocrine system and metabolic processes, while the “distal drugs” are mainly anti-inflammatory and pain relieving. We primarily screened cephalosporin antibiotics and adapalene, currently used as antibacterial and anti-inflammatory drugs [21, 24]. Interestingly, the drug Rubidium Chloride Rb-82 was found, a radioactive substance currently used in PET CT for the diagnosis of coronary heart disease and myocardial infarction [25].

Our study also has some limitations. Firstly, there is a lack of molecular biology experiments as additional validation. Secondly, there is no validation in terms of LTL in patients who clinically develop AD. In conclusion, we verified the causal link between LTL and AD using Mendelian randomization analysis and associated LTL-related lncRNAs. The downstream target genes were obtained by screening through interactions network analysis, which was used as a basis to explore the potential therapeutic drugs that might correspond to the relevant gene clusters. The evidence that LTL is associated with AD at the SNP level was supplemented, and the new gene-drug interaction network was used to screen for possible therapeutic drugs, providing fresh ideas for slowing down AD progression.

## Supplementary Material

Supplementary Tables 1-3

Supplementary Table 4

Supplementary Table 5

Supplementary Table 6

Supplementary Table 7

Supplementary Table 8

Supplementary Table 9

Supplementary Table 10
