# Supplementary material for: Study of the role of leukocyte telomere length-related lncRNA NBR2 in Alzheimer’s disease
Source: Aging (Albany NY). 2024 Sep 16;16(18):12593–607. doi: 10.18632/aging.206107 (PMC11466486; doi:10.18632/aging.206107)
Supplement: Supplementary Tables 1-3 [file aging-16-206107-s001.pdf]

SUPPLEMENTARY TABLES

Supplementary Table 1. IVs associated with LTL on AD.

IVs associated with LTL on AD			
SNP	beta	se	p
rs10112752	0.036245368586056	0.335960595885184	0.914086302744282
rs1023767	0.0318330876610498	0.598202328717915	0.957560936273678
rs10845387	-2.23439804521506	0.770868499879615	0.00374896940811539
rs111527438	0.1226420207984	0.808320154808	0.879404184404874
rs115610405	0.135653125047028	0.331921070657474	0.682766504041688
rs11579626	-0.67653853842701	0.635972285930905	0.287425566351911
rs11646283	-1.03276164498491	0.633135927317576	0.102851350253984
rs11866592	-0.0888565421036374	0.395789941163395	0.82236492450604
rs12369950	-0.725930392584741	0.777176784832986	0.350272515749782
rs12613375	0.299590252043144	0.760846716847368	0.693758967833938
rs1291143	0.432281913149277	0.275809968471748	0.117040392785504
rs12941945	-0.631233574145798	0.497076375695793	0.204122915970736
rs13129697	-0.228596347825633	0.621621569699291	0.713065633921282
rs13230646	-0.839831534225546	0.628136900332993	0.181216141950711
rs1332941	-0.291483776011881	0.509999966268047	0.56763578614708
rs137901416	-0.353778125262467	0.340121648626422	0.298269407866343
rs139795227	-0.00912506310327856	0.734918273251148	0.990093374787841
rs144204502	-0.438618984202677	0.433917038694891	0.312094907111989
rs181647350	-0.914382859633444	0.345861126355731	0.00819844323220483
rs182059586	-0.211057336184845	0.657071988141656	'0.748052127615715
rs1907702	-0.519608790891665	0.769743554453666	0.49964944245128
rs1980240	0.105908226509547	0.745852568573192	0.887083011457418
rs1985369	0.669705295816193	0.465126151917485	0.149913853461556
rs2056726	0.147247860566561	0.498332026017415	0.767626134727134
rs2293607	-0.175791281699995	0.117004673984947	0.132986205123189
rs2303262	0.144742056923011	0.239692079898186	0.545932293003809
rs28502153	-0.0549658376581634	0.45240063767391	0.903296484974332
rs3093888	0.144358322298347	0.737626939057053	0.844839964712432
rs35640778	-0.589704286884422	0.170306915828354	0.000534988207818243
rs35671754	0.714915646701244	0.829696721453537	0.388875194050772
rs3891167	-0.290018592562576	0.278978713760175	0.298538569329448
rs4435700	-0.308159027472066	0.211939422991374'	0.145947475094368
rs4498805	0.741224448277236	0.629774507033154	0.23920827482734
rs4530278	-0.11801644199059	0.70674935941438	0.867381962574147
rs4724	0.0975753234171041	0.279984255974105	0.727462540965029
rs4743037	-1.23284685697873	0.762271434828446	0.105806388376798
rs4758644	-1.18132171212357	0.643015661549952	0.0661864946541454
rs61748181	-0.500385615307278	0.521266249933256	0.3370841662439
rs6590343	-1.51702332287106	0.795494585870592	0.056518121731675
rs6669563	-0.44839645574255	0.524620452345387	0.39271370915957
rs66731853	-1.21970213478748	0.567512463437407	0.031617942178875
rs6776756	-0.53846106593193	0.555723139539896	0.332576305186575
rs73581419	0.247682240388448	0.662445036765026	0.708485409074085
rs76219171	-0.413438596483427	0.564119745961388	0.463624575693405
rs762810	-0.108553589522824	0.491309870506245	0.825133467053132

rs76666449	0.268208322473189	0.528839065080898	0.612039504295859
rs7705526	-0.445782206505743	0.142292531483644	0.00173113540311976
rs7790856	-0.0192834938678268	0.238335937959602	0.935514417689642
rs78491606	-0.421827731636853	0.561317277459934	0.452354161193265
rs79228077	-0.554057619040488	0.76235760281677	0.467367680235681
rs80324517	0.151612587526323	0.559452787897053	0.786389819706275
rs8102497	1.09352745001804	0.641690413812528	0.0883557653618294
rs8105767	0.237753801447695	0.319336341283376	0.456559089212273
rs869785	1.87629640573512	0.677902038118029	0.00564362956915102
rs871134	1.16439596808499	0.530562251542741	0.0281890491407611
rs932002	0.0533718458002945	0.324472234345309	0.869346854618042
rs939916	0.13349415552803	0.424216473992432	0.75300156033797
rs9419958	-0.318804296586339	0.170001746112199	0.0607514454952407
rs9923119	1.66453409680992	0.674964337571699	0.0136590626994769

Supplementary Table 2. Mendelian randomization of exposures on the risk for AD.

Mendelian randomization of exposures on the risk for AD							
**Method**	**nsnp**	**beta**	**se**	**pval**	**OR**	**or_lci95**	**or_uci95**
MR Egger	59	-0.284772361	0.096200533	0.004473661	0.7521855	0.6229273	0.9082648
Weighted median	59	-0.204963971	0.07786336	0.008479532	0.8146767	0.6993694	0.948995
Inverse variance weighted	59	-0.192330809	0.057355096	0.000798442	0.8250339	0.73731	0.923195
Simple mode	59	0.022117346	0.159358624	0.890097059	1.0223637	0.7480948	1.397186
Weighted mode	59	-0.266739507	0.083141208	0.002175884	0.7658725	0.6507067	0.9014211

Supplementary Table 3. Pleiotropy and heterogeneity of the causal association between LTL and AD.

Heterogeneity			
**Method**	**Q**	**Q_df**	**Q-p-val**
MR Egger	86.80831	57	0.006663209
Inverse variance weighted	88.98135	58	0.005524221
**Pleiotropy**			
**Method**	**p-val**		
egger_intercept	0.237224		
