# Supplementary material for: Study of the role of leukocyte telomere length-related lncRNA NBR2 in Alzheimer’s disease
Source: Aging (Albany NY). 2024 Sep 16;16(18):12593–607. doi: 10.18632/aging.206107 (PMC11466486; doi:10.18632/aging.206107)
Supplement: Supplementary Table 8 [file aging-16-206107-s006.pdf]

**Supplementary Table 8. The list of genes associated with GJA1 in AD brain tissue.**

| gene_name1 | gene_name2 | cor_r | p-value |
|---|---|---|---|
| GJA1 | ADD3 | 0.802371206904213 | 1.17341269039321E-21 |
| GJA1 | CSRP1 | 0.820254711108708 | 2.61773616426948E-23 |
| GJA1 | SLC39A12 | 0.801549080433322 | 1.38449947933484E-21 |
| GJA1 | GABRG3 | -0.637275526922121 | 1.1080578580304E-11 |
| GJA1 | GFRA2 | -0.607471408 | 1.71921683018262E-10 |
| GJA1 | GJA1 | 1 | 0 |
| GJA1 | GOT1 | -0.608856007508237 | 1.52315537236684E-10 |
| GJA1 | ICA1 | -0.602400596892852 | 2.66545497842034E-10 |
| GJA1 | IDH3G | -0.626270516361077 | 3.15525095511253E-11 |
| GJA1 | ATP1A2 | 0.824295131116415 | 1.04551338281366E-23 |
| GJA1 | NOTCH2 | 0.808104375749031 | 3.62158286681252E-22 |
| GJA1 | PAX6 | 0.822685726261586 | 1.51115845882501E-23 |
| GJA1 | GRAMD1C | 0.816757790187561 | 5.68767509823794E-23 |
| GJA1 | PLSCR4 | 0.83129683399707 | 2.0132995105433E-24 |
| GJA1 | RASGRF1 | -0.601698028693054 | 2.83070247178229E-10 |
| GJA1 | GRAMD3 | 0.829356830850272 | 3.20169444474726E-24 |
| GJA1 | BEND5 | -0.614344090684302 | 9.37105215524573E-11 |
| GJA1 | CC01 | -0.615299465425736 | 8.60296422575356E-11 |
| GJA1 | HSPB3 | -0.636926806041838 | 1.14615294142954E-11 |
